# Dopamine: A New Player in the Pathogenesis of Diabetic Retinopathy?

**DOI:** 10.3390/ijms252313196

**Published:** 2024-12-08

**Authors:** Marianthi Ntikoudi, Theofano Myrto Farmaki, Konstantinos Tziomalos

**Affiliations:** First Propedeutic Department of Internal Medicine, Medical School, Aristotle University of Thessaloniki, AHEPA Hospital, 54636 Thessaloniki, Greece; marianntikoudi@gmail.com (M.N.); mifarmaki1@gmail.com (T.M.F.)

**Keywords:** diabetes mellitus, diabetic retinopathy, dopamine, pathogenesis, microvascular complications, blindness

## Abstract

Diabetic retinopathy (DR) is a leading cause of blindness. The pathogenesis of diabetic retinopathy is multifactorial and incompletely understood. Accordingly, treatment options are limited. Recent data suggest that dopamine might play a role in the development and progression of DR. In the present review, we discuss these data and comment on the potential role of dopamine modulation in the management of this devastating microvascular complication of diabetes mellitus.

## 1. Introduction

Diabetic retinopathy is one of the most common microvascular complications of diabetes mellitus, affecting more than one-fourth of all diabetic patients [1,2]. Importantly, approximately 5% of patients with diabetes mellitus have vision-threatening diabetic retinopathy [1]. Patients with type 1 diabetes mellitus are at a higher risk of developing diabetic retinopathy compared to those with type 2 diabetes mellitus. Several factors can influence the likelihood and severity of diabetic retinopathy, including hypertension, obesity, smoking, the duration of diabetes, and how well the diabetes is controlled [1,2].

The classic lesions of diabetic retinopathy are microaneurysms, hemorrhages, hard exudates (lipid deposits), cotton wool spots, intraretinal microvascular abnormalities, venous changes, and neovascularization, which eventually culminate to vision impairment and blindness [3]. Retinal panphotocoagulation and antiVEGF injections have reduced the percentage of blindness by more than 90%. However, current treatment has limited efficacy and a high cost and requires further research to improve its effectiveness [4].

## 2. Pathogenesis of Diabetic Retinopathy

The pathogenesis of diabetic retinopathy is incompletely understood and involves multiple mechanisms such as inflammation, activation of the immune system, oxidative stress and accumulation of advanced glycation end products, activation of the polyol, protein kinase C and hexosamine pathways, mitochondrial dysfunction, histone modification and DNA methylation, and imbalance of local production of neurotrophic factors lead to retinal degeneration [5,6,7].

Inflammation is crucial in the pathogenesis of diabetic retinopathy. Elevated levels of inflammatory markers such as interleukin-1β, -6, -8, and -17A, and tumor necrosis factor-α are linked to the severity of diabetic retinopathy. In a hyperglycemic environment, these markers, along with others like monocyte chemoattractant protein-1 are unregulated, promote retinal endothelial cell dysfunction and neovascularization [8]. Proteins like nucleotide-binding leucine-rich repeat family pyrin domain containing 3 and thioredoxin-interacting protein activate inflammatory pathways and cause retinal cell death [9]. Chronic inflammation, therefore, accelerates the progression to proliferative diabetic retinopathy.

Oxidative stress plays a key role in the pathogenesis of diabetic retinopathy. In patients with diabetes, there is an increase in reactive oxygen species and a decrease in antioxidants such as superoxide dismutase and glutathione. This imbalance between oxidants and antioxidants contributes significantly to the complications associated with diabetes. In the context of diabetic retinopathy, elevated levels of reactive oxygen species cause damage to retinal tissues and cells, leading to apoptosis and the progression of the disease [10]. More specifically, excess glucose, catalyzed by aldose reductase, uses nicotinamide adenine dinucleotide phosphate hydrogenase as an electron donor to produce sorbitol, a highly hydrophilic compound that cannot easily penetrate lipid membranes, leading to an increase in cell osmotic pressure and damage to retinal capillary permeability. In addition, sorbitol is converted to fructose by sorbitol dehydrogenase, consuming more nicotinamide adenine dinucleotide phosphate hydrogenase, which is crucial for the glutathione synthesis. Consequently, the epithelial cells lose their resistance to oxidative stress [10].

The increased oxidative stress and production of reactive oxygen species impairs mitochondrial function, damaging the mitochondrial DNA. Mitochondrial DNA is a histone-free DNA, which means it is more vulnerable to free radical damage, although it is complexed with proteins in nucleoid bodies, which offer some protection. In addition, high glucose can produce a large amount of reactive oxygen species by increasing the electron flux of the electron transport chain and decreasing the activity of complex III, leading directly to mitochondrial DNA’s damage. Furthermore, reactive oxygen species increase the expression of two matrix metalloproteinases, 2 and 9, which enter the mitochondria leading to the release of cytochrome C and accelerating capillary apoptosis [11]. It is proved that retinal mitochondria in diabetic retinopathy have a decreased expression of mitofusin-2 (a fusion protein of the outer mitochondrial membrane and an increased expression of dynamin-related fission protein), which damages the mitochondrial fusion–fission machinery. The result of those mechanisms is the breakdown of mitochondrial membrane and cellular apoptosis [12].

Recent studies have shown that there are also epigenetic modifications in diabetes and especially DNA methylation, which is one of the important molecular mechanisms in epigenetics. The diabetic environment facilitates mitochondrial DNA methylation in retinal mitochondria through the key enzyme responsible for methylation, DNA methyltransferase-1. More specifically, DNA methyltransferase-1 in diabetic retinal cells is significantly elevated, leading to hypermethylation of mitochondrial DNA primarily to D-loop region, the region with transcription and replication elements. Due to hypermethylation, some mitochondrial DNA-encoded genes that are important for the electron transport chain system are silenced leading to further leakage of electrons generating superoxide radicals [13].

A recent study reveals that oxidative stress and mitochondrial dysfunction, through which diabetes-induced damage to the retina occurs, remain irreversible even after glucose levels are normalized [14]. While strict glucose control is undeniably crucial for preventing the progression of diabetes and its complications, there is a critical need to develop effective treatments that can protect the retina and fight the phenomenon of hyperglycemic memory. Unraveling the pathways that underpin the development and progression of diabetic retinopathy might enable the identification of novel therapeutic targets. In this context, accumulating data suggest that dopamine might represent an important player in the development of diabetic retinopathy.

In this review, we summarize the role of dopamine in the pathogenesis of diabetic retinopathy and the therapeutic potential of modulating this pathway in patients with diabetic retinopathy.

## 3. Role of Dopamine in the Retina

**Figure 1:**
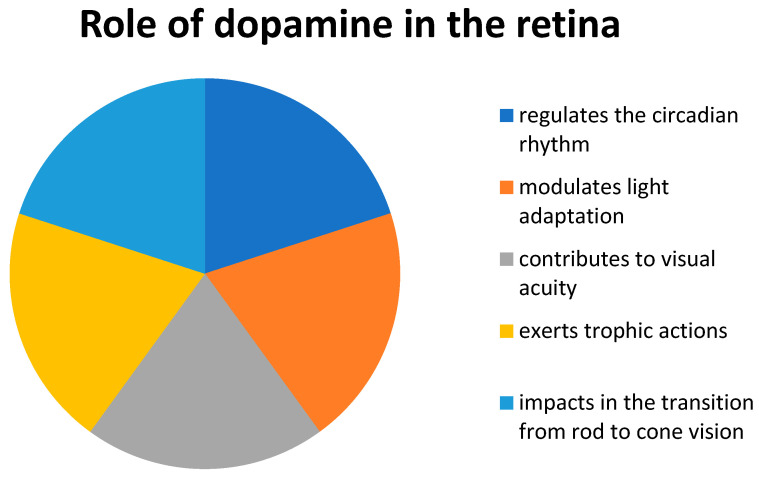
Role of dopamine in the retina.

Dopamine is a fundamental neurotransmitter in the human body that plays a critical role in various functions, including motor behavior, cognition, and emotional state. Numerous diseases such as Parkinson’s disease, Huntington’s chorea, tardive dyskinesia, and schizophrenia are associated with altered dopamine levels, proving the significant role of dopamine in human health [15]. In the retina of all vertebrates, dopamine functions primarily as a neuromodulator in the circuitry (Figure 1) [16,17]. Dopamine is widely recognized as a pivotal neurotransmitter in light adaptation and circadian rhythm [18,19]. Dopamine levels peak at dawn as the visual system transitions from rod-dominant to cone-dominant vision. This increase in dopamine enhances the efficiency of information processing through cone-based vision, improving visual clarity in daylight conditions. At the same time, dopamine temporarily reduces the sensitivity of rod-based vision, aligning visual function with daytime needs [15,19]. Furthermore, the role of dopamine in growth, development, and cell death, collectively known as trophic functions of the retina, is also being increasingly recognized [15,19].

In the retina, dopamine is released by dopaminergic amacrine cells through the action of tyrosine hydroxylase, which converts tyrosine to L-dopa, further metabolized into dopamine [16,17]. Amacrine cells are one of the multiple retinal neurons that contribute to visual processing and have more than 30 different subtypes [20]. Dopamine effects in the retina are mediated through its action on specific dopamine receptors categorized into D1- and D2-like receptors, which activate different intracellular signaling pathways [15]. In particular, the D1-like receptor family includes the D1 and D5 receptors, which activate the guanosine nucleotide-binding protein (G protein) adenylate cyclase and produce cyclic adenosine monophosphate as a second messenger [15]. They also activate phospholipase C and, in this way, induce intracellular calcium release, which causes not only the production of many proteins such as calcium-dependent protein kinase C, but also the modulation of neurotransmitter release of exocytosis [15]. In addition, these receptors regulate the electrochemical gradient through Na^+^-K^+^-ATPase [15]. The D2-like receptor family includes the D2, D3, and D4 receptors, which inhibit the activation of adenylate cyclase and, as a result, the production of cyclic AMP [21]. The interaction between D1- and D2-like receptors and their distribution among different cell types in the retina allows for the complex modulation of visual processing, adapting the visual system to a wide range of environmental light conditions and contributing to visual acuity and performance [15].

## 4. Dopamine and Diabetic Retinopathy

The connection between dopamine and diabetic retinopathy has been a subject of research for many years (Table 1). An early study published in 1985 in diabetic rats reported a significant decrease in the concentration of dopamine in the retina starting from the third week after the development of hyperglycemia [22]. This decrease appeared to be due to the increased efflux of dopamine from the retinal tissue, whereas the uptake of dopamine by the retina did not differ between diabetic animals and controls [22]. Interestingly, rodents subjected to photic stimulation showed reduced retinal release of dopamine, suggesting that the depletion of dopamine may contribute to retinal dysfunction [22]. It is well established that several visual abnormalities, including color vision disturbances and contrast sensitivity deficits, are associated with diabetic retinopathy [23].

More recent research using rats with streptozotocin-induced diabetes mellitus also demonstrated that a dysfunction in the dopaminergic system of the retina might be implicated in the pathogenesis of diabetic retinopathy [24]. More specifically, a greater than 50% reduction in tyrosine hydroxylase-positive cells, a subtype of amacrine cells that are responsible for dopamine production in the retina, was noted in the retina of diabetic rats [24]. Moreover, in rats subjected to light, dopamine levels in the retina were lower in diabetic animals than in controls [24]. In contrast, a more recent study reported increased dopamine levels in the retina of a rat model of type 2 diabetes mellitus [25]. These animals also showed a substantial delay in flicker and oscillatory potential implicit times [25]. It is possible that the increase in dopamine levels in this animal model of diabetes mellitus might represent a protective mechanism against the development of diabetic retinopathy [25].

Another study in rats and mice reported that the induction of diabetes mellitus reduces retinal dopamine levels, which coincide with the development of a visual deficit [26]. In addition, the administration of l-DOPA, a dopamine precursor, ameliorated retinal and visual function [26]. Moreover, acute treatment with D1- or D4-receptor agonists improved spatial frequency threshold and contrast sensitivity, respectively [26]. It has also been reported that the development of diabetes mellitus after the administration of streptozotocin in rats is rapidly followed by increased apoptosis of dopaminergic amacrine cells in the retina [27].

In another study in diabetic rodents, a significant correlation between low dopamine levels in the retina and a delay in oscillatory potentials in an electroretinogram was observed [28]. Interestingly, administration of l-DOPA preserved oscillatory potential implicit time [28]. In diabetic, retina-specific tyrosine hydroxylase knockout mice, a delay in oscillatory potentials was observed compared with diabetic, wild-type mice, and was restored with l-DOPA treatment [28].

In a more recent study, mice with streptozotocin-induced diabetes mellitus were treated with insulin to achieve normoglycemia, thereby mimicking the phenomenon of metabolic memory, in which diabetic retinopathy progresses although glucose levels have normalized [14]. In this model, oxidative stress, mitochondrial membrane potential collapse and fission, adherent junction disassembly, and vascular leakage were observed in the retina of mice despite the presence of normoglycemia, possibly reflecting the effects of hyperglycemic memory [14]. Intravitreal injection of levodopa ameliorated these abnormalities and prevented pericyte degeneration, acellular capillary and pericyte ghost generation, as well as endothelial apoptosis [14]. Similar findings were reported in human retinal endothelial cells after treatment with dopamine [14]. The study concludes that dopamine can significantly inhibit the persistent generation of intracellular and mitochondrial reactive oxygen species induced by hyperglycemia in both human retinal endothelial cells and mouse retinas. These elevated reactive oxygen species levels persisted even after the glucose levels were normalized. However, when dopamine was injected, there was a significant inhibition of reactive oxygen species production, proving its antioxidative effect [14]. Additionally, it was demonstrated that dopamine suppresses mitochondrial membrane collapse by reducing dynamin-related protein-1 phosphorylation and mitofusin expression in the retinas of mice with hyperglycemia, functioning as a protection for mitochondrial homeostasis. It was also proved that dopamine has a notable inhibitory effect on vascular endothelial growth factor-induced changes in vascular function, particularly concerning vascular permeability and adherence junction stability [14].

Another study suggests that starting L-DOPA treatment upon the first signs of visual and retinal dysfunction, rather than at the onset of diabetes, could effectively reverse the course of retinopathy and offer neuroprotection [29]. Electroretinography and optomotor response (OMR) are two techniques that could contribute to the early detection of these abnormalities, upon which the following study is conducted. In rats with streptozotocin-induced diabetes mellitus, a reduction in spatial frequency thresholds and oscillatory potential implicit times was observed 6 weeks after the development of diabetes mellitus [29]. However, treatment with l-DOPA starting from 3 weeks after the administration of streptozotocin preserved both spatial frequency thresholds and oscillatory potential implicit times [29]. The study also reveals the potential reversing effect of L-DOPA on the damaged diabetic retinas, highlighting its neuroprotective properties.

Emerging evidence suggests that these findings in preclinical models might also apply to humans. In a recent pilot study, patients with type 2 diabetes mellitus but without clinically detectable diabetic retinopathy were randomized to receive either low- (25 mg carbidopa/100 mg L-DOPA) or high-dose dopamine (50 mg carbidopa/200 mg L-DOPA) for 2 weeks [30]. Patients with diabetes mellitus had significant oscillatory potential implicit time delays detected through electroretinography (ERG) compared with age-matched non-diabetic, control subjects [30]. Oscillatory potentials are produced inside the amacrine cells, and processing visual information and their implicit time is prolonged in diabetes, indicating a delay in visual stimulation. More importantly, treatment with dopamine restored these times to control values, and these improvements persisted after a 2-week washout period [30].

**Table 1:** Major findings of studies regarding the association between dopamine and diabetic retinopathy.

Decreased concentration of dopamine in diabetes mellitus [22,24]
Reduction in amacrine cells in diabetes mellitus [24,27]
Amelioration of retinal and visual function after administration of L-DOPA [26]
Prevention of pericyte degeneration, acellular capillary and endothelial apoptosis after treatment with L-DOPA [14]
Inhibition of vascular endothelial growth factor production in diabetes mellitus [14]
Improvement of oscillatory potential implicit times and spatial frequency thresholds after treatment with L-DOPA [29]
Antioxidative effect of dopamine [14]

## 5. Neurodegeneration in Diabetic Retinopathy

Over the past 20 years, it has become clear that diabetic retinopathy impacts not only the vascular system but also neuronal tissue. While the relationship between vascular damage and neuronal degeneration remains uncertain—whether they are interrelated or independent processes—the occurrence of neurodegeneration in diabetic retinopathy is well established [31]. The process involves the excessive loss of neurons by apoptosis (specifically in the inner retina), along with structural and biochemical changes that may impact the nervous system. Several experts studying neurodegeneration in diabetic retinopathy have found that both cholinergic and dopaminergic amacrine cells, as well as photoreceptors, are affected.

Given that optical coherence tomography findings in diabetic retinopathy also appear in other neurodegenerative diseases like Alzheimer’s and Parkinson’s, it is worthwhile to examine the pathways involved in these conditions to confirm or refute their presence in diabetic retinopathy. A potentially common observation across these diseases could be elevated levels of certain neurotransmitters that may be toxic to the retina, alongside decreased levels of others that could offer protective effects [31]. The retina utilizes various neurotransmitters, including dopamine, glutamate, gamma-aminobutyric acid, and acetylcholine. Glutamate is released in large amounts, particularly under low-light conditions [31]. In diabetes, glutamate levels become dysregulated, as shown by experimental studies in diabetic models that reveal increased extracellular glutamate levels [32]. This dysregulation induces an excessive influx of intracellular calcium, initiating a cascade of pathological events that ultimately lead to retinal cell death [32]. However, it is unclear whether this increase results from decreased glutamate metabolism or an upregulation of its receptors. Consequently, the potential of targeting glutamate or its receptors for therapy remains uncertain [31]. Regarding gamma-aminobutyric acid, there appears to be a reduction in its levels. This gamma-aminobutyric acid deficiency leads to elevated glutamate levels because the usual negative feedback mechanism through gamma-aminobutyric acid is impaired. Consequently, managing glutamate levels could be effectively addressed by using drugs that target α-amino-3-hydroxy-5-methyl-4-isoxazolepropionic acid (AMPA) receptors, which appear to be modulated by a reduction in Ca^2^⁺ permeability within the receptor channels [31]. Additionally, compounds like berberine, which recent studies suggest may rescue retinal ganglion cells through the activation of GABA-A receptors, could further support neuroprotection [33]. Together, these strategies offer a potential dual approach for regulating excitatory and inhibitory signaling in the retina, promoting overall neuronal health.

The reduction in dopamine in hyperglycemia is relatively more understood. Tyrosine hydroxylase, a key enzyme for dopamine synthesis and a marker for amacrine cells, is notably decreased. The reduction in tyrosine hydroxylase and the associated degeneration of amacrine cells may contribute to the decreased dopamine levels observed in diabetes. The administration of L-dopa, which is used to treat other neurodegenerative diseases, has been shown to improve retinal function in diabetic mice, as measured by the optomotor tracking reflex. Additionally, monoamine oxidase B inhibitors, such as selegiline and rasagiline, have been used alongside dopamine treatments to reduce the metabolism of the neurotransmitter and enhance its availability. Apart from that, selegiline helps to reduce oxidative stress and provides protection to Müller cells, retinal neurons, and spontaneously arising retinal pigment epithelial cells from apoptosis. As previously mentioned, the loss of amacrine cells also impacts the cholinergic system [31]. In diabetes, the production of acetylcholine is reduced. Although acetylcholine and its receptor agonists have demonstrated effectiveness in other retinal diseases, there is currently no evidence supporting their efficacy in diabetic retinopathy [27]. Consequently, the dopaminergic system presents a more promising target for future therapeutic interventions compared to other neurotransmitter systems.

## 6. Diabetic Retinopathy and Parkinson’s Disease

Parkison’s disease is one of the most well-known conditions associated with dysfunction in the dopaminergic system. Numerous studies have simultaneously examined Parkison’s disease and diabetic retinopathy, highlighting their common characteristics [34,35,36,37].

Diabetic retinopathy is increasingly recognized as a progressive neurodegenerative disease just like Parkinson’s disease. Experts propose that these diseases may exhibit similar pathophysiological features, including mitochondrial dysfunction, endoplasmic reticulum stress, chronic inflammation, and neurodegeneration playing a key role in its pathogenesis. These shared pathways could contribute to the development and progression of both conditions, highlighting potential intersections in their pathogenesis and possible avenues for therapeutic strategies [34,35]. However, the relationship between PD and DR is still controversial as there is evidence that indicates a reduced risk of PD in patients with DR [37], while other studies suggest that there is a higher incidence of PD in DM and DR patients [35,36].

Dopamine is a key neurotransmitter in the retina, particularly involved in modulating the activity of amacrine cells. Diabetes-induced loss of amacrine cells leads to a deficiency in retinal dopamine levels. This connection is particularly intriguing because both diabetic retinopathy and Parkinson’s disease share a common feature: dopamine deficiency. In Parkinson’s disease, the deficiency occurs in the brain, while in diabetic retinopathy, it manifests in the retina. This overlap suggests a potential link between the two conditions, emphasizing the broader implications of dopamine dysregulation in diabetes-related complications [34,35]. The proven effectiveness of L-DOPA in treating Parkinson’s disease provides encouraging hope that it might also hold potential in treating diabetic retinopathy.

## 7. Current Treatment of Diabetic Retinopathy

Currently, the suggested treatments of diabetic retinopathy that reduce the progression to blindness include intravitreal injections of anti-vascular endothelial growth factor drugs (ranibizumab, pegaptanib, and aflibercept) that inhibit the abnormal blood vessel growth. Anti-angiogenic therapies have revolutionized the treatment of diabetic retinopathy, with ranibizumab emerging as one of the most effective options. However, vascular endothelial growth factor inhibitors like ranibizumab have a relatively short half-life, necessitating monthly or bimonthly intravitreal injections. The frequent injection schedule can increase the risk of significant side effects, including endophthalmitis [38,39]. Also, other side effects are related to anti-VEGF factors like elevation in intraocular pressure, vitreous hemorrhage, and inflammation, establishing the need for a novel treatment approach [39]. Currently, intravitreal anti-VEGF injections are the main treatments for DME, such as brolucizumab, which has proven its efficacy in improving both visual and morphological outcomes after administration in patients for over 24 weeks [39]. Diabetic macular edema (DME) is a retinal condition resulting from an imbalance between the influx and outflow of fluid, as well as impaired retinal hydraulic conductivity, leading to the accumulation of intraretinal fluid. Retinal hypoxia increases the permeability of the retinal capillaries through the production of VEGF, a key mediator in DME development [39]. However, over 40% of eyes with diabetic macular edema were refractory to anti-VEGF therapies [39].

Corticosteroids, with their anti-inflammatory effect, are used to reduce cytokines involved in diabetic retinopathy. Intravitreal steroids specifically target vascular endothelial growth factor, tumor necrosis factor-α, chemokines, leukostasis, and the phosphorylation of tight-junction proteins. While triamcinolone has shown significant improvements in visual acuity, it was eventually withdrawn due to side effects such as elevated intraocular pressure and cataracts. Currently, FDA-approved options in this category include dexamethasone implants (Ozurdex) and fluocinolone acetonide implants (Iluvien) [39].

Another approved treatment is pan-retinal laser photocoagulation, which used to be the gold standard treatment before the advent of anti-vascular endothelial growth factor therapy. Retinal laser photocoagulation can significantly reduce the risk of visual loss, primarily through the alleviation of macular edema and the regression of neovascularization. In advanced stages of diabetic retinopathy where other treatments are insufficient, vitrectomy is the last surgical intervention that is performed [38,39].

Several innovative treatments for diabetic retinopathy are currently in clinical trials, targeting different aspects of the disease’s pathophysiology. A notable example is squalamine, an anti-angiogenic agent that inhibits not only vascular endothelial growth factor but also other growth factors such as platelet-derived growth factor and basic fibroblast growth factor. In the realm of anti-inflammatory therapies, interleukin-6 inhibitors, interleukin-6 receptor inhibitors (tocilizumab), and integrin inhibitors (luminate) have emerged as a promising novel treatment for diabetic retinopathy. Finally, new laser techniques, like pattern scanning lasers, are currently under investigation to minimize the side effects associated with traditional laser treatments [39,40].

However, these treatments are expensive and carry a risk of complications. Therefore, there is a growing interest in earlier detection and treatment strategies to identify retinal defects before structural vascular changes occur and to explore whether early interventions can prevent the progression of diabetic retinopathy and subsequent vision loss.

## 8. Dopamine’s Role in Early Detection of DR

As mentioned before, numerous clinical trials suggest that dopamine deficiency may contribute to visual dysfunction, and furthermore, normalizing dopamine levels could slow the progression of diabetic retinopathy [22,24]. The main three capabilities of dopamine that highlight its crucial role in diabetic retinopathy are the protective effect in hyperglycemia, the amelioration of visual dysfunction, and neuroprotection. However, L-dopa treatment has demonstrated effectiveness in the early stages of diabetic retinopathy [29], emphasizing the critical importance of early detection and intervention. Several methods for early detection of diabetic retinopathy should be employed in patients without clinical signs of the disease. These early detection methods can be divided into two categories: tests of retinal structure and tests of retinal function. For assessing retinal structure, techniques such as optical coherence tomography, optical coherence tomography angiography, and retinal vascular caliber measurements are commonly used. For evaluating retinal function, methods such as electroretinography, contrast sensitivity, microperimetry, color vision tests, and retinal oximetry are valuable tools [41].

In the context of the early detection of diabetic retinopathy, the potential role of caffeine presents significant interest. Caffeine is known to enhance the release of dopamine and other neurotransmitters [42,43], which could have a beneficial effect on dopamine levels, influencing the progression of retinal damage in diabetes. A recent clinical study investigated the prospective protective effects of moderate-to-high caffeine intake (through coffee or tea consumption) against diabetic retinopathy in individuals with type 2 diabetes [44]. This effect appears to be most relevant in the early stages of diabetes, before other complications have developed. Notably, caffeine intake was associated with a 65% reduction in the risk of developing diabetic retinopathy. However, an experimental study in mice found that caffeine had no impact on the development of diabetic retinopathy, highlighting the need for further research to determine whether caffeine itself offers protective effects or if other compounds, such as antioxidants in coffee or tea, are responsible for this potential benefit [44].

## 9. Conclusions

Accumulating preclinical data suggest that dopamine might play a role in the pathogenesis of diabetic retinopathy. Given the unmet needs regarding the management of this vision-threatening and frequent complication of diabetes mellitus, the efficacy of modulating this pathway might be worth further evaluation in humans. New advances in early detection methods for diabetic retinopathy may unveil a critical treatment window during which neuroprotective agents could be utilized to prevent or delay progression to vision loss. Further research is needed to precisely determine how dopamine deficiency contributes to the progression of diabetic retinopathy. Given that neurodegeneration affecting the retina appears to be primarily driven by deficiencies in dopamine—a key neurotransmitter in the retina—understanding this relationship is crucial for developing targeted treatments.
